# Successfully treatment of penile vitiligo patches and their sexual dysfunction consequences, by suction blister epidermal grafting^☆^

**DOI:** 10.1016/j.abd.2021.05.025

**Published:** 2022-12-23

**Authors:** Moradi Mahmoudreza, Kavoussi Hossein, Kavoussi Reza

**Affiliations:** a Kermanshah University of Medical Science, Kermanshah, Iran; b Hajdaie Dermatolgy Clinic, School of Medicine, Kermanshah University of Medical Sciences, Kermanshah, Iran

Dear Editor,

Vitiligo is a common pigmentary disease with many psycho-social consequences such as sexual dysfunction (SD). In the treatment of refractory vitiligo such as vitiligo lesions in glabrous areas, medical treatment is disappointing. In recent years’ surgical interventions such as autologous non-cultured melanocyte grafting (ANCMG) and suction blister epidermal grafting (SBEG) were developed for the treatment of stable vitiligo.^1^, ^2^ But vitiligo patches on problematic-to-treat areas, such as male genital even with this method may be with poor outcomes.^2^, ^3^

A 32-year-old male was presented with depigmented patches located on the glans penis and associated SD from 58 and 32 months ago respectively. Laboratory examination, including thyroid, showed no abnormal findings. The patient was married 6 years ago, but 28 months later suffered from SD, because he and his wife feared that vitiligo was contagious. He had been subjected to multiple treatments including ANCMG by dermatology and Sexual Disorder Center (SDC) but had no appropriate treatment response. New lesions had not developed in the last 12 months.

We suggested SBEG because of problematic-to-treat areas and not responding to previous treatment. Firstly, anesthetized depigmented patches were abraded. The anterolateral of the leg consider a donor site and used funnel cylinder technique^3^ is in order to harvest grafts. The harvested blister was detached; was then located over the recipient site.

We recommended partial bed rest for 7 days, being very careful when using the toilet, and avoidance of situations that induce penile erection. Complete repigmentation was achieved without any complication after 3 months (Figs. 1–3 ).

**Figure 1:**
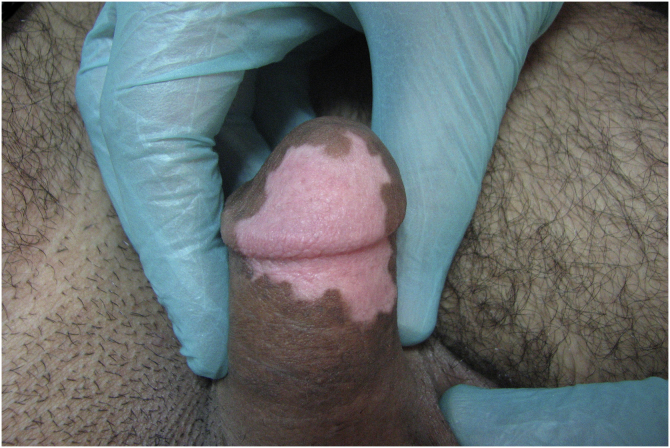
Penile vitiligo.

**Figure 2:**
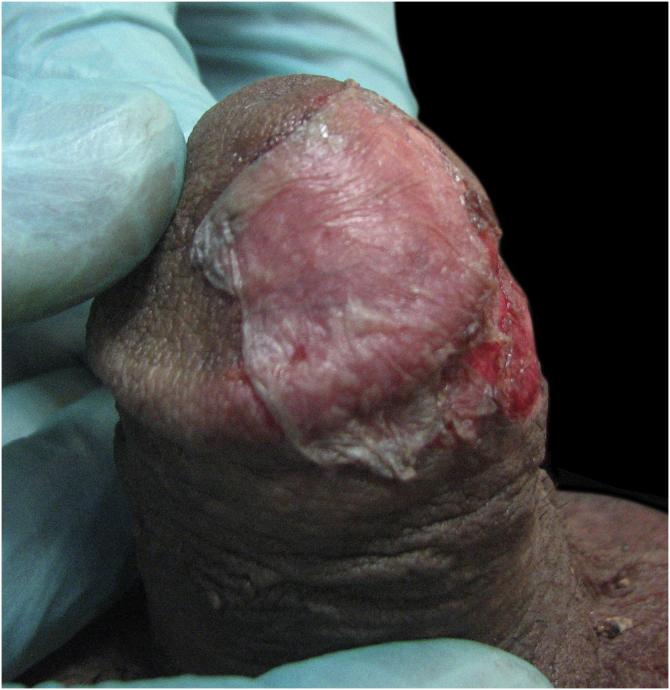
Harvested epidermal graft located over the recipient abraded site.

**Figure 3:**
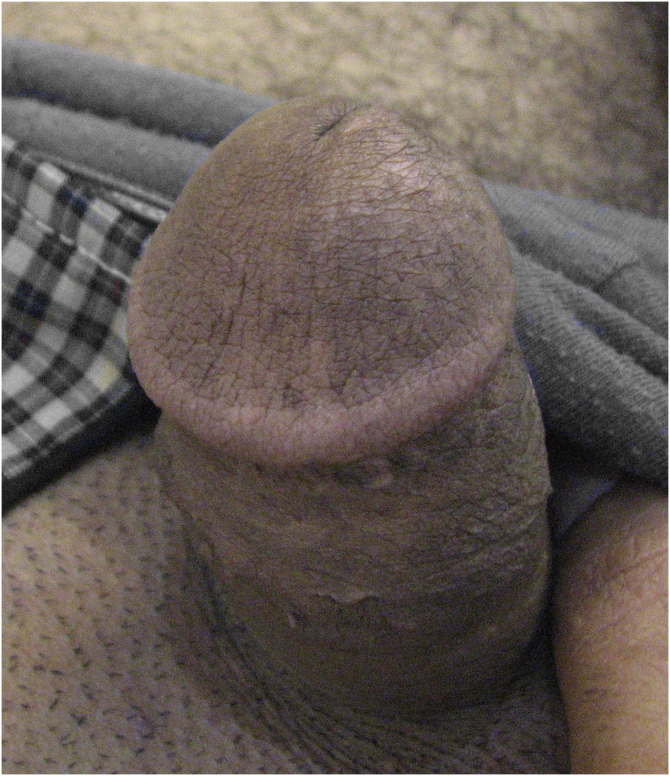
Successful outcome of treated sites after 3 months.

For the management of SD, we referred the patient to SDC. After 12 months, he presented with persistent repigmentation, improvement of SD and pregnancy of his wife.

Sukan and co-workers^4^ demonstrated that chronic skin diseases such as vitiligo have undesirable influences on sexual activity. But other studies showed the presence or absence of genital vitiligo patches had not different effects on sexual functions.^5^

It seems in our patient, SD was consequence of vitiligo, because of was absent of no abnormality finding throughout evaluations at SDC and was induced SD prior to vitiligo.

In limited studies with a few cases of genital vitiligo, that were treated by ANCMG, poor to good repigmentation outcome was obtained.^1^, ^3^

We believe that the failure of surgical interventions in male genital is related to its mobility, change in size, erection, susceptibility to infection and care, especially during the toilet.

SBEG is an efficient method for the management of limited, stable, and resistant vitiligo with variable treatment results.^2^, ^3^ In the SBEG method, fixation of recipient sites is very important in order to achieve an optimal outcome.^2^ It is very difficult to keep male genital in an immobility state.

In the literature review, our patient is the first case, that successfully improved both vitiligo patches and SD through SBEG. We suggest SBEG in stable vitiligo patches on male genital with its SD consequence.

This case report was approved by the Ethics Committee of Kermanshah University of Medical Sciences. The patient signed informed consent.

## Financial support

None declared.

## Authors’ contributions

Mahmoudreza Moradi: Study conception and planning; critical literature review.

Hossein Kavoussi: Approval of the final version of the manuscript; effective participation in research orientation; study conception and planning; manuscript critical review.

Reza Kavoussi: Intellectual participation in propaedeutic and/or therapeutic management of studied cases; preparation and writing of the manuscript.

## Conflicts of interest

None declared.

## References

[1] Ramos M.G., Ramos D.G., Ramos C.G. (2017). Evaluation of treatment response to autologous transplantation of noncultured melanocyte/keratinocyte cell suspension in patients with stable vitiligo. An Bras Dermatol.

[2] Ebrahimi A., Radmanesh M., Kavoussi H. (2015). Recipient site preparation for epidermal graft in stable vitiligo by a special fraise. An Bras Dermatol..

[3] Dellatorre G., Bertolini W., Castro C.C.S. (2017). Optimizing suction blister epidermal graft technique in the surgical treatment of vitiligo. An Bras Dermatol..

[4] Sukan M., Maner F. (2007). The problems in sexual functions of vitiligo and chronic urticaria patients. J Sex Marital Ther..

[5] Yucel D., Sener S., Turkmen D., Altunisik N., Sarac G., Cumurcu H.B. (2021). Evaluation of the Dermatological Life Quality Index, sexual dysfunction and other psychiatric diseases in patients diagnosed with vitiligo with and without genital involvement. Clin Exp Dermatol..

